# Convergent hub pathways targeted by IAV, SARS-CoV-2, and RSV in type II alveolar epithelial cells: molecular mechanisms and therapeutic implications

**DOI:** 10.3389/fimmu.2026.1781447

**Published:** 2026-03-11

**Authors:** Kaixuan Zhang, Sudi Zhu, Mengyu Zhang, Henggui Hu, Shuguo Qin, Huihui Li, Pingping Zhao, Yuanyuan Xu

**Affiliations:** Department of Clinical Laboratory, Wanbei Coal Electric Group General Hospital, Suzhou, China

**Keywords:** type II alveolar epithelial cells, SARS-CoV-2, signal transduction, immune evasion, molecular interaction, respiratory syncytial virus, host-directed therapy, influenza A virus

## Abstract

Type II alveolar epithelial cells (AEC2s) maintain surfactant homeostasis, support distal-lung repair, and contribute to antiviral innate defense. Influenza A virus (IAV), SARS-CoV-2, and respiratory syncytial virus (RSV) use distinct entry receptors, yet severe disease is repeatedly marked by AEC2 dysfunction, alveolar barrier failure, and dysregulated inflammation. We synthesize cross-virus evidence for convergence on a small set of host hubs: innate sensing and interferon signaling, mitochondria-centered immunometabolism and oxidative stress, post-translational signaling modules, barrier and surfactant programs, and regulated cell-death checkpoints. We summarize structural and post-translational mechanisms by which viral proteins disrupt pattern recognition receptor (PRR)–mitochondrial antiviral signaling protein (MAVS) signaling, couple mitochondrial injury to weakened antiviral responses, and bias epithelial fate toward inflammatory lytic injury. Where AEC2-specific evidence is incomplete, especially for integrated PANoptosis-like programs, we label these elements as working models and highlight validation needs. We compare model systems used to study AEC2 infection, including ALI cultures, organoids, lung-on-chip platforms, and single-cell or network analyses. Finally, we discuss host-directed therapeutic opportunities along the cascade, separating near-term approaches from longer-term platform strategies such as targeted protein degradation and targeted nanodelivery, and noting constraints in distal-lung delivery, onset kinetics, and safety. This AEC2-centered convergence framework supports mechanism-driven interpretation of severe viral pneumonia and guides broader-spectrum intervention concepts.

## 1 Introduction

**Figure 1:**
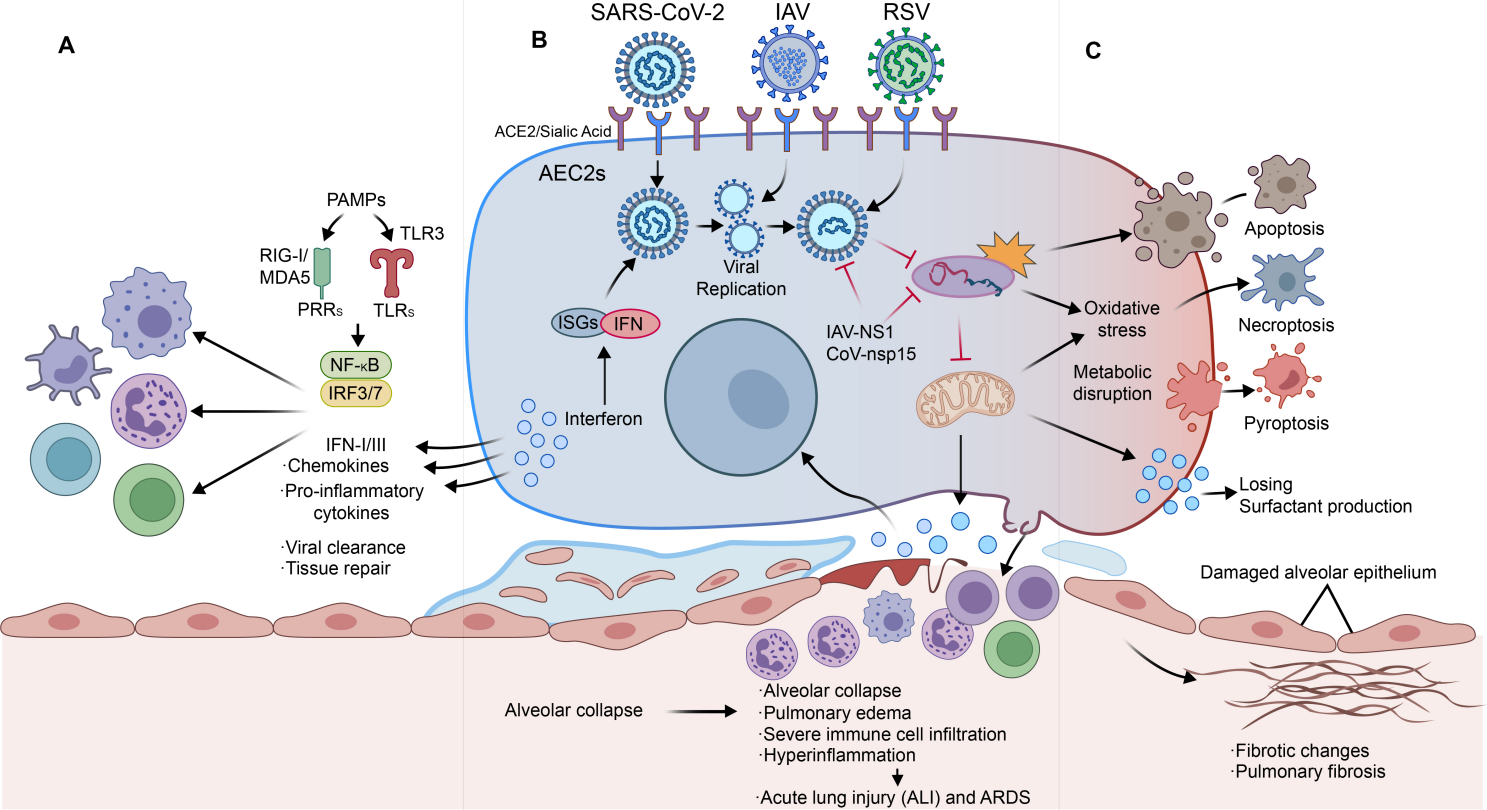
AEC2-centered pathogenic cascade from viral entry and innate sensing to epithelial dysfunction and severe pneumonia outcomes. **(A)** Immune recognition and output: viral PAMPs activate PRRs (e.g., RIG-I/MDA5 and TLRs). This induces NF-κB and IRF3/7 signaling. Interferons and inflammatory mediators shape immune-cell recruitment and tissue repair. **(B)** AEC2 infection and replication: IAV, SARS-CoV-2, and RSV enter AEC2s via distinct receptors and replicate in AEC2s. IFN signaling induces ISGs, forming a core antiviral program. **(C)** Stress and injury outcomes: viral replication and viral antagonism promote oxidative stress and metabolic disruption. Surfactant production declines. Cell-death programs (apoptosis, necroptosis, pyroptosis) contribute to barrier injury, edema, ALI/ARDS, and fibrotic remodeling in severe/prolonged disease. Symbols: → indicates direction of effect/flow; red inhibitory bars indicate viral antagonism. AEC2s, type II alveolar epithelial cells; ACE2, angiotensin-converting enzyme 2; PAMPs, pathogen-associated molecular patterns; PRRs, pattern recognition receptors; RIG-I, retinoic acid-inducible gene I; MDA5, melanoma differentiation-associated protein 5; TLRs, Toll-like receptors; NF-κB, nuclear factor kappa B; IRF3/7, interferon regulatory factor 3/7; IFN, interferon; ISGs, interferon-stimulated genes; ALI, acute lung injury; ARDS, acute respiratory distress syndrome; IAV, influenza A virus; RSV, respiratory syncytial virus.

Acute respiratory viral infections represent a major global public health challenge. Influenza A Virus (IAV), SARS-CoV-2, and Respiratory Syncytial Virus (RSV) are the primary pathogens responsible for lower respiratory tract infections and severe complications, such as Acute Lung Injury (ALI) and Acute Respiratory Distress Syndrome (ARDS) (1–3). Although these viruses exhibit significant differences in taxonomy, genomic structure, and epidemiology, they all elicit similar alveolar epithelial injury and dysregulated inflammatory responses following infection (4–7). This suggests that they may drive disease severity by perturbing shared, critical cellular and molecular pathways within the lung. Human type II alveolar epithelial cells (AEC2s) are the central guardians of alveolar structural and functional homeostasis, responsible for synthesizing pulmonary surfactant, differentiating into type I cells for repair, and serving as the first line of defense in pulmonary innate immunity (8–10). Advances in single-cell sequencing and imaging technologies have increasingly clarified the role of AEC2s in viral infections (9). However, existing reviews largely focus on single viruses, lacking a systematic comparison and integration of the convergent mechanisms employed by different viruses specifically within AEC2s. Here, we construct a multi-level analytical framework centered on AEC2s to systematically compare the pathogenic mechanisms of IAV, SARS-CoV-2, and RSV. As illustrated in **Figure 1**, while these viruses invade via different receptors, they trigger convergent pathogenic cascades that ultimately result in the destruction of the alveolar epithelial barrier and the onset of ALI/ARDS. Beyond the chronological pathology, we aim to reveal the underlying molecular logic network, focusing on: (1) structural interactions between viral proteins and host defense factors; (2) dynamic regulation of post-translational modifications in immune and stress signaling and their targeted disruption; and (3) cross-talk and synergistic amplification among different stress pathways.

Based on this framework, we advance an integrative hypothesis: severe viral pneumonia arises when distinct viral factors convergently perturb a small set of AEC2 functional hubs that couple immune surveillance, metabolic support, barrier integrity, and epithelial repair. We test this hypothesis by comparing IAV, SARS-CoV-2, and RSV across shared hubs and by mapping mechanistic links from molecular interactions to alveolar barrier failure. We then discuss host-directed therapeutic opportunities and practical constraints for translating hub-based concepts into interventions.

Scope, literature selection, and definitions. This is a narrative, mechanism-centered review. We prioritized peer-reviewed primary studies that directly examined AEC2s or AEC2-enriched distal-lung epithelial models. These include primary AEC2s, ALI and organoid systems with alveolar epithelial signatures, lung-on-chip platforms, and *in vivo* or multi-omic datasets with AEC2 annotation. We also included studies that provided mechanistic links between viral factors and host signaling or cell-fate control. In this review, a hub denotes a host process that integrates multiple upstream perturbations and exerts broad downstream control over epithelial function. Convergence denotes mechanistically distinct viral factors producing comparable perturbations of the same hub, leading to shared AEC2-relevant outcomes.

## 2 Invasion and recognition: structural basis of receptor interaction and spatiotemporal control

### 2.1 Differentiated receptor binding and conformational activation mechanisms

Viral invasion commences with the specific binding of viral surface proteins to host receptors. The Receptor Binding Domain (RBD) of the SARS-CoV-2 Spike protein undergoes significant conformational rearrangement upon binding to Angiotensin-Converting Enzyme 2 (ACE2), transitioning from a closed (down) to an open (up) state, thereby exposing protease cleavage sites and the fusion peptide (11–14). Cryo-EM studies have revealed that key aromatic residues on the RBD (e.g., Tyr449) form a tight hydrophobic interface with the complementarity-determining region of ACE2, which is crucial for high-affinity binding (15). In contrast, the Hemagglutinin (HA) protein of IAV binds to sialic acid (SA) receptors at the terminals of cell membrane glycoproteins or glycolipids via its globular head domain. The characteristics of amino acids at positions 226 and 228 in the HA receptor-binding pocket determine its preference for α2,3- or α2,6-linked SA, thereby influencing host range and tissue tropism (16). The Fusion (F) protein of RSV undergoes complex proteolytic processing to form a pre-fusion conformation composed of trimers. Upon binding to cell surface receptors (e.g., heparan sulfate proteoglycans or integrins), its hydrophobic fusion peptide is exposed and inserted into the target cell membrane, triggering an irreversible transition to a highly stable post-fusion conformation, which is the core driver of membrane fusion (17–19). AEC2s are central maintainers of alveolar homeostasis and represent an important distal-lung target in severe viral pneumonia. Early infection dynamics and immunopathology involve multiple compartments, including airway epithelium, immune cells, and endothelium. Their relative contributions can differ by virus and disease stage. Single-cell transcriptomic analysis confirms that ACE2 expression in AEC2s is significantly higher than in other lung epithelial cell types and is upregulated by interferon stimulation, providing a molecular basis for the alveolar tropism of SARS-CoV-2 (20). For IAV, the specific glycosylation profile of sialic acid receptors on the AEC2 surface (e.g., the ratio of α2, 3- to α2,6-linked SA) may determine the infection efficiency of different avian or human strains (21). Nucleolin, a receptor for RSV, is enriched on the plasma membrane of AEC2s and may interact with Surfactant Protein A (SP-A), which is specifically expressed by AEC2s, to synergistically mediate viral endocytosis (22). The unique distribution and regulation of these receptors in AEC2s collectively shape the primary terrain for viral invasion.

### 2.2 Molecular assembly and post-translational regulation of PRR signaling

Following the entry of viral nucleic acids into the cytoplasm, their recognition by Pattern Recognition Receptors (PRRs) involves precise molecular assembly and post-translational modifications. RIG-I, a RIG-I-like receptor (RLR), specifically recognizes the 5’-triphosphate group and short double-stranded regions of viral RNA via its C-terminal repressor domain (CTD) (23). Upon binding, RIG-I undergoes a conformational change, releasing its tandem Caspase Recruitment Domains (CARDs). Subsequently, the E3 ubiquitin ligase TRIM25 catalyzes the formation of K63-linked polyubiquitin chains at the K172 site of the RIG-I CARD domain (24). This ubiquitination serves as a molecular scaffold, promoting CARD-CARD homotypic interactions between RIG-I and the mitochondrial antiviral signaling protein (MAVS), thereby initiating the prion-like polymerization of MAVS on the outer mitochondrial membrane (25). The MAVS polymer acts as a massive signaling platform, recruiting and activating the TBK1/IKKϵ kinase complex, which in turn phosphorylates and activates the transcription factors IRF3/7 (26). This series of events strictly depends on dynamic changes in protein subcellular localization and an ordered relay of post-translational modifications. As terminally differentiated epithelial cells, AEC2s possess a unique configuration of innate immune signaling networks adapted to their physiological functions. Studies indicate that the RIG-I/MAVS signaling axis in AEC2s is tightly coupled with their mitochondrial functional state (27). Given that AEC2s require substantial phospholipid synthesis for surfactant production, their mitochondria are metabolically active with high membrane potential, which may provide a favorable microenvironment for MAVS polymerization. Furthermore, the induction of Interferon-Stimulated Genes (ISGs) in AEC2s exhibits unique temporal patterns and subtype preferences, such as a rapid response to type III interferons, which may help limit excessive systemic inflammation while controlling the virus, thereby protecting the fragile alveolar gas exchange structures (28).

### 2.3 Structural disruption and precision intervention of PRR signaling by viruses

**Figure 2:**
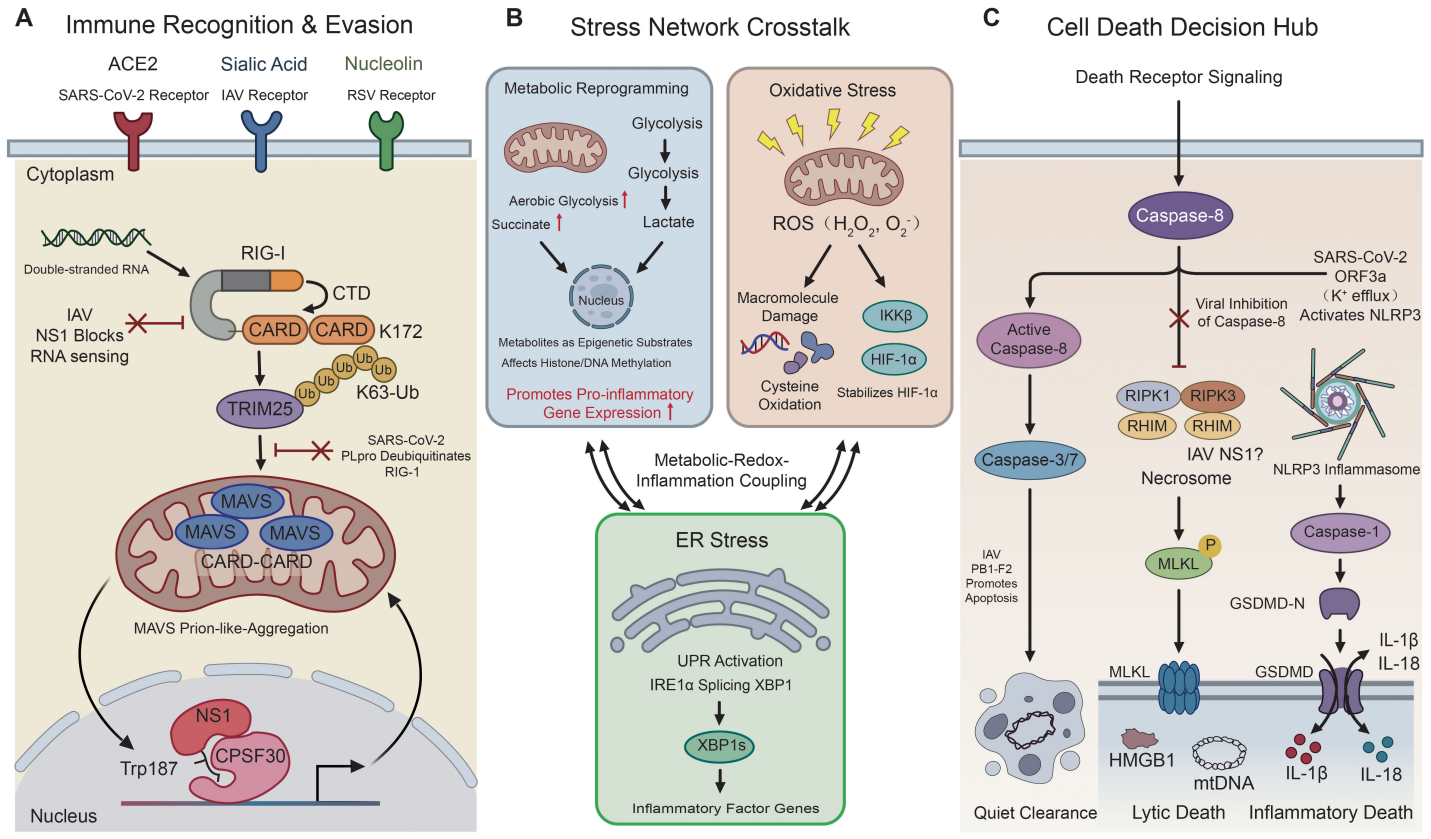
Convergent AEC2 hub processes targeted across viruses: immune evasion, stress-network crosstalk, and cell-death decision control. **(A)** Immune recognition and evasion: entry receptors and representative viral interference with PRR–interferon signaling in AEC2s. SARS-CoV-2 uses ACE2, IAV uses sialic acid, and RSV uses nucleolin. Viral antagonists disrupt RIG-I/MAVS pathway assembly and signaling propagation, weakening interferon-driven antiviral programs. **(B)** Stress-network crosstalk: metabolic remodeling, oxidative stress (ROS), and ER stress/UPR reinforce each other and promote inflammatory gene programs. **(C)** Cell-death decision hub: caspase-8-centered checkpoints connect apoptosis, necroptosis, and inflammasome/pyroptosis outputs. The balance shapes regulated clearance versus lytic inflammatory death. Symbols: arrows indicate propagation; red inhibitory bars and “X” marks indicate inhibition/antagonism. AEC2, type II alveolar epithelial cell; ACE2, angiotensin-converting enzyme 2; PRR, pattern recognition receptor; PAMPs, pathogen-associated molecular patterns; RIG-I, retinoic acid-inducible gene I; MDA5, melanoma differentiation-associated protein 5; TLR3, Toll-like receptor 3; TLRs, Toll-like receptors; NF-κB, nuclear factor kappa B; IRF3/7, interferon regulatory factor 3/7; IFN, interferon; MAVS, mitochondrial antiviral-signaling protein; TRIM25, tripartite motif-containing protein 25; CPSF30, cleavage and polyadenylation specificity factor 30 kDa subunit; CTD, C-terminal domain; PLpro, papain-like protease; ROS, reactive oxygen species; ER, endoplasmic reticulum; UPR, unfolded protein response; IRE1α, inositol-requiring enzyme 1α; XBP1s, spliced X-box binding protein 1; HIF-1α, hypoxia-inducible factor 1α; IKKβ, inhibitor of nuclear factor kappa B kinase subunit beta; NLRP3, NLR family pyrin domain containing 3; RHIM, RIP homotypic interaction motif; RIPK1/3, receptor-interacting protein kinase 1/3; MLKL, mixed lineage kinase domain-like protein; GSDMD, gasdermin D; IL, interleukin; HMGB1, high mobility group box 1; IAV, influenza A virus; RSV, respiratory syncytial virus.

To counter this sophisticated host defense system, viruses have evolved antagonist proteins that directly target key molecular interaction interfaces. The Non-Structural Protein 1 (NS1) of IAV inserts its conserved tryptophan residue (e.g., Trp187 in human IAV) into the hydrophobic pocket of the host pre-mRNA 3’-end processing factor CPSF30 via its effector domain. This competitively inhibits the binding of CPSF30 to its natural substrates with nanomolar affinity, globally blocking the maturation of numerous host mRNAs, including those encoding interferons (29, 30). SARS-CoV-2 utilizes its Papain-Like Protease (PLpro) to exert deubiquitinase and de-ISGylating activities, specifically removing K63-linked ubiquitin chains or ISG15 modifications from signaling molecules like RIG-I and STING, thereby removing host immune activation signals at the post-translational level (31–34). Additionally, SARS-CoV-2 Non-Structural Protein 15 (Nsp15), a metal ion-dependent endoribonuclease, cleaves viral RNA via conserved histidine residues in its active center, reducing the accumulation of long double-stranded RNAs that activate MDA5 (35). To visualize the complexity of this virus–host antagonism, **Figure 2** systematically outlines and compares the key immune evasion proteins of IAV (e.g., NS1), SARS-CoV-2 (e.g., PLpro, Nsp15), and RSV (e.g., NS1/NS2) and their targets, revealing how three viruses converge on the core objective of suppressing host antiviral immunity through differentiated molecular viral antagonist proteins weapons (34, 36, 37). Viral intervention in AEC2 immune signaling may have profound consequences beyond immune evasion. IAV NS1 binding to CPSF30 globally inhibits host mRNA 3’-end processing. In AEC2s, this may impede the maturation of mRNAs encoding critical alveolar homeostatic proteins (such as Surfactant Proteins B and C), thereby directly disrupting alveolar function early in infection, rather than merely suppressing interferons (38). Similarly, the deubiquitinating activity of SARS-CoV-2 PLpro on RIG-I may disturb the delicate balance between mitochondrial metabolism and immune signaling in AEC2s, as MAVS aggregation is likely regulated by adjacent mitochondrial membrane proteins (39). Thus, viral targeting of AEC2s can blunt innate alarm signaling and weaken the cellular programs that sustain surfactant secretion and barrier maintenance.

### 2.4 Synthesis: core functional hubs and cross-virus vulnerabilities

**Figure 3:**
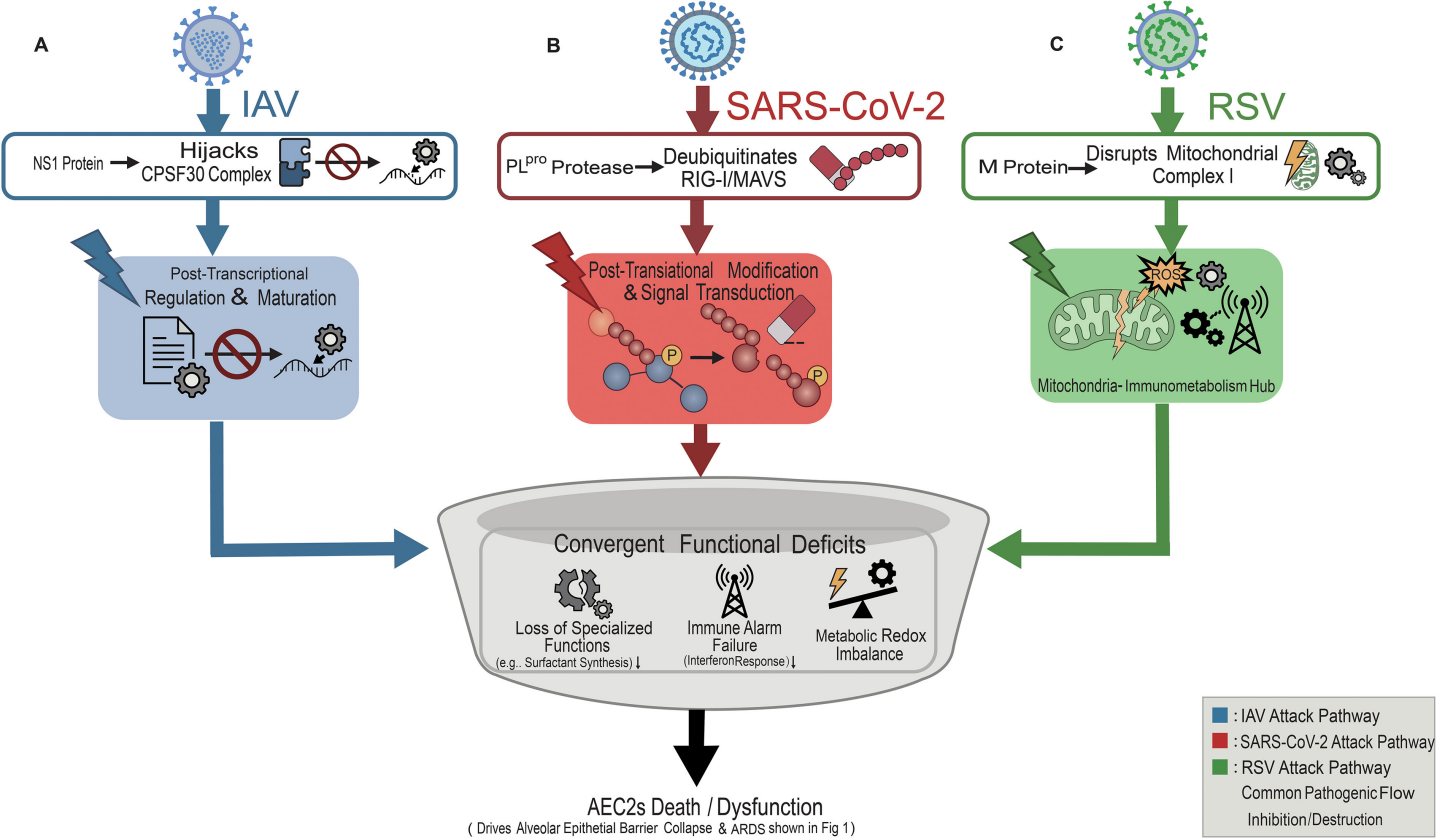
Virus-specific antagonists converge on core AEC2 functional modules and produce shared deficits. **(A)** IAV: NS1 interaction with the CPSF30 complex disrupts post-transcriptional regulation and protein maturation programs in AEC2s. **(B)** SARS-CoV-2: PLpro-mediated deubiquitination perturbs RIG-I/MAVS-associated post-translational signaling and antiviral signal transduction. **(C)** RSV: M protein–linked disruption of mitochondrial complex I promotes mitochondrial stress/ROS, impairing mitochondria–immunometabolism coupling. Distinct upstream perturbations converge on functional deficits, including reduced specialized functions (e.g., surfactant synthesis), immune alarm failure (reduced interferon response), and metabolic–redox imbalance. These changes promote AEC2 dysfunction and downstream barrier injury. Symbols: colored arrows indicate virus-associated pathways (blue, IAV; red, SARS-CoV-2; green, RSV). ↓ indicates impairment/loss. AEC2s, type II alveolar epithelial cells; IAV, influenza A virus; RSV, respiratory syncytial virus; PLpro, papain-like protease; CPSF30, cleavage and polyadenylation specificity factor 30 kDa subunit; RIG-I, retinoic acid-inducible gene I; MAVS, mitochondrial antiviral-signaling protein; ROS, reactive oxygen species; ARDS, acute respiratory distress syndrome.

Summarizing the invasion and early interference mechanisms above, we propose three interlinked functional hubs that are repeatedly perturbed across IAV, SARS-CoV-2, and RSV (**Figure 3**). First is a post-transcriptional regulation and maturation hub. AEC2s require rapid stress responses and sustained surfactant production. These functions depend on coordinated RNA processing and protein maturation (40, 41). Second, a post-translational modification and signal-transduction hub. Ubiquitination, phosphorylation, and deubiquitination modules shape PRR-IFN signaling dynamics. Viral proteins can rewire these checkpoints and blunt antiviral propagation. Third, a mitochondria-immunometabolism hub. AEC2 mitochondria provide bioenergetic support for secretory programs. They also scaffold innate immune signaling, including MAVS platforms. Viral perturbations can couple metabolic injury to interferon suppression (42). Together, disruption of these hubs yields convergent functional deficits. It promotes AEC2 dysfunction. It can also bias cell-fate programs discussed in later sections (43, 44).

## 3 Cellular stress and dysfunction: crosstalk in the metabolic-oxidative-inflammatory network

### 3.1 Coupling of metabolic reprogramming and immune epigenetics

Viral infection induces profound metabolic remodeling in AEC2s, the impact of which extends far beyond providing raw materials for viral replication. Virus-driven aerobic glycolysis leads to the accumulation of metabolic intermediates. The enhancement of the pentose phosphate pathway not only provides ribose-5-phosphate for nucleotide synthesis but also generates substantial NADPH to maintain redox balance and lipid synthesis (45–48). More importantly, changes in key metabolite levels directly affect the activity of epigenetic modifying enzymes. Elevated Acetyl-CoA, a substrate for histone acetyltransferases, promotes histone acetylation at pro-inflammatory gene promoters, enhancing their transcription (49, 50). Alterations in the ratio of tricarboxylic acid (TCA) cycle intermediates, such as α-ketoglutarate to succinate, systematically reprogram the cellular response to immune stimuli at the epigenetic level by influencing the activity of ten-eleven translocation (TET) family DNA demethylases and histone demethylases, including the attenuation of ISG induction (51, 52).

### 3.2 Oxidative stress as signal amplifier and damage executor

The burst of Reactive Oxygen Species (ROS) induced by viral infection is not only a direct cause of macromolecular oxidative damage but also acts as a critical intracellular signaling messenger. Superoxide anions produced by electron leakage from the mitochondrial electron transport chain are converted to hydrogen peroxide (H_2_O_2_) by superoxide dismutase. H_2_O_2_ can alter the conformation and activity of signaling proteins by oxidatively modifying thiol groups on key cysteine residues; for instance, the activities of IKKβ and ASK1 are regulated by their redox states (53–55). Additionally, ROS stabilizes Hypoxia-Inducible Factor 1α (HIF-1α); even under normoxic conditions, H_2_O_2_ inhibits prolyl hydroxylase activity, leading to HIF-1α accumulation, which in turn transcriptionally upregulates glycolytic genes and pro-inflammatory cytokines (56). The RSV Matrix (M) protein has been shown to localize to mitochondria and inhibit Complex I activity, directly inducing mitochondrial ROS production, while its non-structural proteins synergistically block immune signals, linking oxidative stress to interferon suppression (48, 57, 58). This metabolic-redox-transcriptional coupling constitutes a positive feedback loop that amplifies the inflammatory response. This cross-virus mitochondrial targeting reflects a shared logic. Mitochondria are bioenergetic organelles. They are also immune-signaling hubs. Whether through IAV-associated loss of membrane potential or RSV M protein-linked respiratory chain inhibition, a shared outcome is impaired MAVS platform function with increased mitochondrial ROS. This coupling links metabolic injury to weakened antiviral signaling.

### 3.3 ER stress and the immunomodulatory role of the UPR

The massive synthesis of viral proteins and membrane remodeling caused by viral replication impose immense pressure on the Endoplasmic Reticulum (ER) of AEC2s, triggering the Unfolded Protein Response (UPR) (59, 60). Among the three main branches of the UPR, the activation of Inositol-Requiring Enzyme 1 α (IRE1α) is particularly critical. Activated IRE1α possesses endoribonuclease activity, enabling the non-canonical splicing of X-box Binding Protein 1 (XBP1) mRNA to produce transcriptionally active XBP1s. XBP1s not only upregulates ER chaperone expression to restore homeostasis but also directly binds to and regulates the promoters of various inflammatory cytokine genes, including IL-6 and TNF-α (59, 61). Meanwhile, sustained UPR can globally inhibit protein translation via the PERK pathway while selectively upregulating transcription factors such as ATF4, which drives pro-apoptotic gene expression (62). Thus, ER stress becomes a key hub connecting viral replication, inflammation amplification, and cell death decisions.

### 3.4 Summary: AEC2s link epithelial injury to alveolar immune activation

AEC2s are not merely targets of viral attack but are the core initiators and regulators of local alveolar immunity. Following infection, AEC2s actively recruit and activate resident Alveolar Macrophages (AMs) and recruited monocytes by releasing a series of alarmins and cytokines (e.g., IL-33, GM-CSF, CCL2) (63–65). Crucially, the mode of viral-induced AEC2 death directly determines the nature of this dialogue: non-lytic apoptosis favors immunotolerant clearance, while lytic necroptosis or pyroptosis releases massive amounts of Damage-Associated Molecular Patterns (DAMPs, such as ATP and HMGB1), polarizing AMs towards a pro-inflammatory M1 phenotype and activating the NLRP3 inflammasome, thereby amplifying the inflammatory cascade (66, 67). Therefore, viral disruption of AEC2 stress and death programs can alter both the initiation and the magnitude of alveolar immune responses. This shift can turn regulated defense into self-amplifying inflammation.

### 3.5 Summary: integrated stress networks bias AEC2 fate toward inflammatory death

Viral-induced metabolic reprogramming, oxidative stress, and ER stress are not independent events; they form a self-amplifying pathogenic network (68). For example, the IRE1α pathway activated by ER stress can splice XBP1 to promote inflammation; accumulated ROS not only damages macromolecules but also serves as a signaling molecule to activate pro-death kinases like ASK1 (69, 70). More importantly, sustained and intense stress signals deplete the cell’s adaptive reserves, rendering survival pathways (such as the adaptive branches of the UPR) ineffective. At this point, if apoptotic checkpoints (such as Caspase-8, inhibited by viral proteins) are bypassed, stress signals directly pave the way for more lytic necroptosis or pyroptosis. Thus, the uncontrolled stress network is the key driver determining the cell’s trajectory toward inflammatory death (71).

## 4 Integrated regulation of cell death: molecular checkpoints and viral manipulation

### 4.1 Molecular switches of apoptosis, necroptosis, and pyroptosis

Programmed cell-death pathways share checkpoints that determine whether apoptosis, necroptosis, or pyroptosis predominates. Caspase-8 is a central decision node. When active downstream of death receptors, caspase-8 initiates extrinsic apoptosis through effector Caspases-3/7. It also suppresses necroptosis by cleaving RIPK1 and RIPK3 (62). When caspase-8 activity is inhibited by viral factors or constrained by cellular context, RIPK1 and RIPK3 can oligomerize via RHIM interactions to form the necrosome. RIPK3 then phosphorylates MLKL, which oligomerizes and translocates to the plasma membrane, causing membrane permeabilization and necroptosis (72). Pyroptosis is driven by inflammasome activation. Inflammasomes activate caspase-1. Caspase-1 cleaves GSDMD to release its pore-forming N-terminus. Caspase-1 also processes pro-IL-1β and pro-IL-18 into mature cytokines (73).

### 4.2 Precision viral intervention in death signaling networks

Viruses manipulate cellular death fate by targeting key nodes in these pathways to facilitate their own dissemination or evade immune clearance. The PB1-F2 protein of IAV can form non-selective channels on the mitochondrial membrane, inducing membrane potential collapse and Cytochrome c release, thereby potently activating the intrinsic apoptotic pathway (71, 74, 75). However, the NS1 protein of certain IAV strains can indirectly attenuate apoptosis by inhibiting RIG-I signaling. SARS-CoV-2 ORF3a protein has been confirmed to possess ion channel activity, specifically promoting potassium efflux—a classic trigger for NLRP3 inflammasome activation, thereby steering the infection towards pro-inflammatory pyroptosis (76, 77). More strategically, some viruses mimic host protein functions; for example, viral FLICE-inhibitory proteins encoded by certain herpesviruses contain death effector domains that bind to host FADD or Caspase-8, directly inhibiting death receptor signaling (78).

### 4.3 Mutual amplification of death pathways and inflammatory signals

Cell death is not merely the endpoint of injury but a potent source of inflammatory stimulation. Physiologically apoptotic cells are typically cleared quietly by macrophages via eat-me signals. However, when clearance mechanisms are overwhelmed or when cells undergo lytic death such as necroptosis or pyroptosis, massive amounts of DAMPs (e.g., HMGB1, ATP, mtDNA) are released (79, 80). These DAMPs can be recognized by PRRs on neighboring cells, further activating the NLRP3 inflammasome or NF-κB pathway, forming a death–inflammation positive feedback loop that drastically exacerbates local and systemic inflammation (81, 82). Specific viral-induced death modes (e.g., the preference of SARS-CoV-2 for pyroptosis) may be a significant cause of the severe cytokine storm (83–85).

### 4.4 PANoptosis in viral pneumonia

Recent work suggests that during viral infection, features of apoptosis, necroptosis, and pyroptosis can co-occur and be coordinated through PANoptosome assemblies, a concept termed PANoptosis (86–89). In influenza models, ZBP1 has been linked to coupled activation of RIPK3- and caspase-8–associated pathways and to engagement of inflammasome components, supporting integrated activation across death programs (90–94, 97–100). In SARS-CoV-2 studies, ORF3a has been associated with ion flux and NLRP3 activation and has also been linked to apoptotic signaling, which may create conditions for overlapping death-pathway activation (95, 96). These observations support the view that mixed death-pathway activation can contribute to inflammatory tissue injury in severe viral pneumonia. However, the composition and cell-type specificity of these complexes, particularly in primary AEC2s, remain incompletely defined. We therefore treat AEC2-centered convergence as a working framework, as described in Section 4.5.

### 4.5 A working convergence model: PANoptosis-like inflammatory lytic death in AEC2s

**Figure 4:**
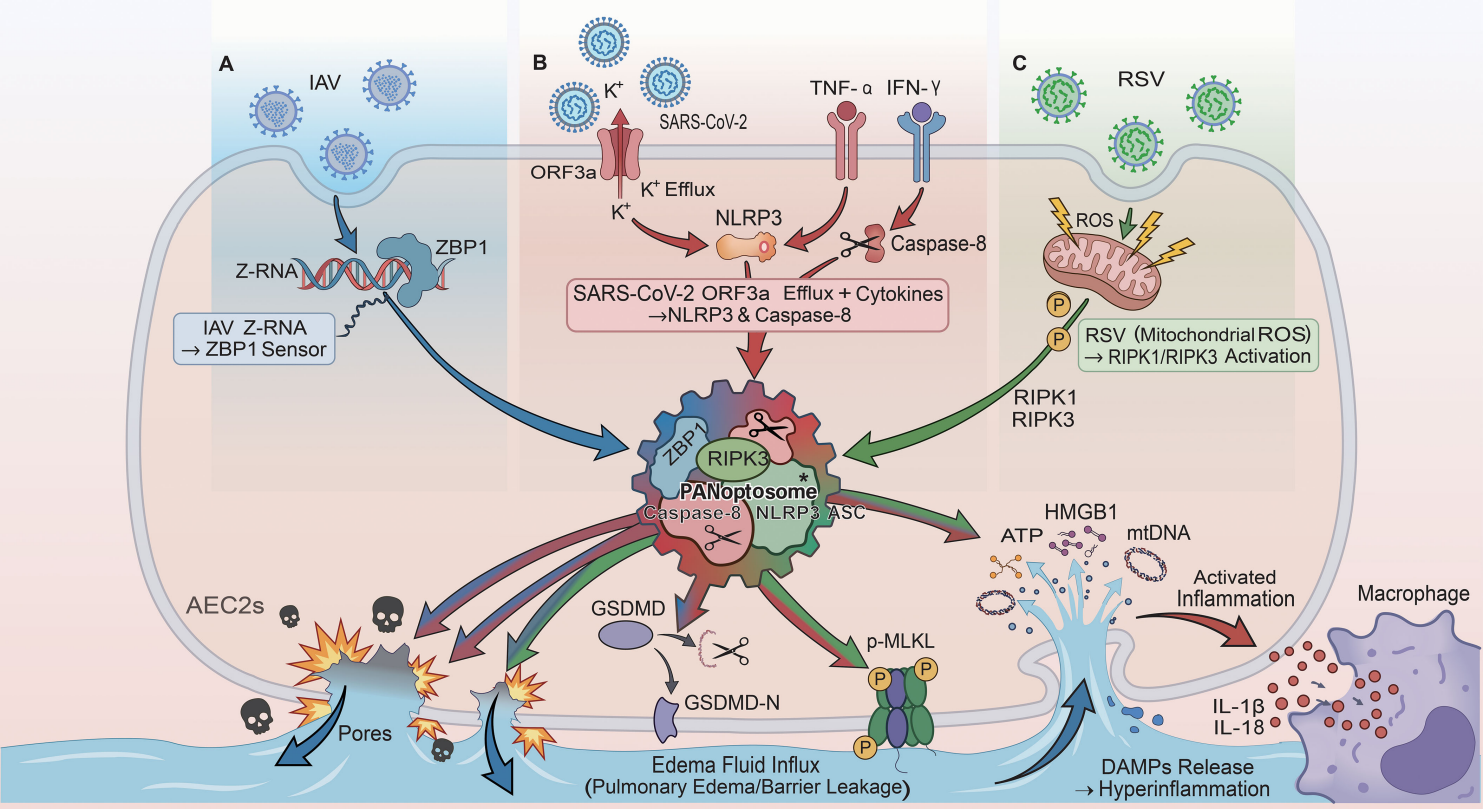
Working model of PANoptosis-like inflammatory lytic injury in AEC2s driven by convergent inputs from IAV, SARS-CoV-2, and RSV. **(A)** IAV-related upstream cue: IAV Z-RNA is shown as a trigger for ZBP1-linked sensing, providing one route into a PANoptosis-like signaling hub in AEC2s. **(B)** SARS-CoV-2-related upstream cue: SARS-CoV-2 ORF3a-associated K^+^ efflux, together with inflammatory cytokine inputs (e.g., TNF-α and IFN-γ), is shown converging on caspase-8/NLRP3-associated signaling. **(C)** RSV-related upstream cue: RSV-associated mitochondrial ROS is shown as a trigger linked to RIPK1/RIPK3 activation. These virus-associated inputs converge on a PANoptosome-like core (center) that integrates ZBP1-, RIPK3-, caspase-8-, and inflammasome-related signaling and can engage membrane pore formation and lytic injury pathways (including GSDMD cleavage and MLKL activation). The downstream consequences shown include barrier leakage/edema fluid influx, DAMP release (ATP, HMGB1, mtDNA), macrophage activation, and amplification of local inflammation (including IL-1β and IL-18).Symbols: blue/red/green arrows indicate virus-associated inputs (IAV/SARS-CoV-2/RSV, respectively); black arrows indicate downstream effects/flow; circled “P” indicates phosphorylation; scissors indicate proteolytic cleavage. Interpretation note: this figure is a mechanistic synthesis/working model for AEC2-centered convergence. Some links are better supported than others in AEC2s and are presented here to organize testable hypotheses rather than to imply equal levels of direct validation. Asterisks mark components/interactions that require direct AEC2-specific validation. AEC2s, type II alveolar epithelial cells; IAV, influenza A virus; RSV, respiratory syncytial virus; ZBP1, Z-DNA-binding protein 1; ORF3a, open reading frame 3a; ROS, reactive oxygen species; RIPK1/3, receptor-interacting protein kinase 1/3; NLRP3, NLR family pyrin domain containing 3; ASC, apoptosis-associated speck-like protein containing a CARD; GSDMD, gasdermin D; GSDMD-N, N-terminal fragment of gasdermin D; MLKL, mixed lineage kinase domain-like protein; p-MLKL, phosphorylated MLKL; DAMPs, damage-associated molecular patterns; ATP, adenosine triphosphate; HMGB1, high mobility group box 1; mtDNA, mitochondrial DNA; TNF-α, tumor necrosis factor alpha; IFN-γ, interferon gamma; IL, interleukin.

Across IAV, SARS-CoV-2, and RSV, multiple upstream signals relevant to necroptosis, pyroptosis, and apoptosis have been reported. Integrated PANoptosis frameworks have been proposed in viral infection contexts (101). Direct AEC2-specific evidence demonstrating complete assembly of a PANoptosome complex remains limited. We therefore describe PANoptosis in AEC2s as a working convergence model rather than an established endpoint. If PANoptosis-like inflammatory lytic death occurs in AEC2s, two consequences are expected. First, pore-forming executioners such as MLKL and GSDMD can breach epithelial membranes, promoting barrier failure and protein-rich alveolar edema (102–106). Second, lytic death promotes DAMP release, which can amplify macrophage and neutrophil activation and inflammasome signaling, reinforcing local inflammatory loops (107–109). Based on the upstream cues summarized above, we propose a convergence hypothesis in AEC2s. Initiating inputs may differ. IAV involves ZBP1-linked sensing. SARS-CoV-2 may involve ORF3a-associated ion flux and cytokine signaling. RSV may involve mitochondrial ROS and DAMP-linked signaling. These pathways may intersect at shared nodes such as RIPK3 and caspase-8 (110). This intersection could enable a PANoptosome-like core that co-activates MLKL and GSDMD (111, 112) (**Figure 4**). This model provides a coherent logic for shared inflammatory lytic injury patterns. It also highlights a key knowledge gap that requires direct validation in AEC2-relevant models.

### 4.6 Summary: a convergent map from molecular attack to pathological endpoints

To integrate the molecular network from immune evasion (Section 2), intrinsic stress amplification (Section 3), and cell-death decision control including PANoptosis-like programs (Section 4), **Table 1** summarizes representative viral factors and convergent consequences of IAV, SARS-CoV-2, and RSV targeting core AEC2 functional hubs. Although the viral factors differ, their pathogenic logic is partially shared. This convergence provides a mechanistic basis for alveolar barrier failure and ALI/ARDS discussed in the next section.

**Table 1:** Convergent molecular pathogenic mechanisms of IAV, SARS-CoV-2, and RSV in human AEC2s.

Functional hub	IAV mechanism	SARS-CoV-2 mechanism	RSV mechanism	Convergent consequence	References
Immune Sensing	NS1 binds TRIM25, blocking RIG-I ubiquitination; directly sequesters dsRNA.	PLpro deubiquitinates RIG-I/MDA5; Nsp15 cleaves viral RNA to evade MDA5.	M protein induces MAVS degradation; blocks RIG-I/MAVS interaction.	Immune silence: blockade of type I/III IFN production, allowing early viral replication in a low-alarm state.	(24, 31, 35, 37, 77)
Mitochondrial Homeostasis	PB1-F2 localizes to the inner mitochondrial membrane, dissipating membrane potential.	ORF3a causes mitochondrial damage via K+ efflux; ORF9b targets TOM70.	M Protein inhibits Complex I, leading to ATP depletion and mitochondrial fragmentation.	Metabolic–immune decoupling: energy failure, disassembly of MAVS signaling platforms, and ROS burst.	(48, 50, 70, 75)
Gene Expression & Maturation	NS1 hijacks CPSF30 complex, blocking host mRNA polyadenylation and nuclear export.	Nsp1 plugs the ribosomal mRNA channel; Nsp16 modifies viral mRNA cap to evade recognition.	M protein translocates to the nucleus to globally suppress host gene transcription.	Functional shutdown: impaired synthesis and maturation of pulmonary surfactants (SP-B/C), driving alveolar collapse (atelectasis).	(30, 38, 40, 115)
Cell Death Decision	ZBP1 senses Z-RNA to initiate PANoptosis-like signaling; NS1 inhibits caspase-8.	Cytokines (TNF/IFN) synergistically drive PANoptosis-like signaling; ORF3a activates NLRP3.	Activates a multimodal cell-death network, involving necroptosis/pyroptosis.	Barrier collapse & storm: lytic death releases DAMPs, driving a death–inflammation positive feedback loop leading to ARDS.	(76, 91, 96, 97, 101)

## 5 Alveolar barrier collapse: cascade amplification from molecular injury to tissue failure

Virus-induced oxidative stress, especially H_2_O_2_, can act as a signal that promotes barrier disintegration. Redox signaling can sustain Src-family kinase activity by transiently inhibiting protein tyrosine phosphatases such as PTP1B. Src activation can increase MLCK activity, driving actin-myosin contraction. This mechanical tension can separate junctional complexes. Oxidative modifications can also target junction proteins. For example, oxidation of cysteine residues in tight-junction components such as occludin can promote conformational change, dissociation, endocytosis, and degradation. Together, these steps connect ROS accumulation to junction disruption and increased epithelial permeability.

### 5.1 Structural dissociation of tight and adherens junctions

Alveolar epithelial barrier function depends on the integrity of intercellular junction complexes. Tight junctions are centered on the Claudin and Occludin protein families, which form selective permeability barriers through trans-interactions with homologous proteins on adjacent cells via their extracellular loop domains (113). Adherens junctions maintain tissue adhesion through calcium-dependent homophilic binding of E-cadherin extracellular domains. Viral infection and associated inflammatory factors can activate MLCK, leading to MLC phosphorylation and cytoskeletal contraction, physically pulling junction proteins away from cell boundaries (114). Furthermore, certain viral proteases or host inflammation-associated proteases (e.g., Matrix Metalloproteinases) can specifically cleave junction proteins. Studies show that SARS-CoV-2 infection correlates with the downregulation of Claudin-7, potentially compromising alveolar epithelial barrier properties (20).

### 5.2 Disorders in surfactant synthesis, assembly, and secretion

The biogenesis of lamellar bodies in AEC2s is a highly complex and precisely regulated process. Hydrophobic surfactant proteins B and C rely heavily on molecular chaperones and specific lipid environments for correct folding and transport. ER stress and oxidative stress triggered by viral infection can directly impair these processes. Protein Disulfide Isomerase activity is affected by redox states, while correct folding of surfactant proteins requires the formation of specific intramolecular disulfide bonds (115). Moreover, virus-induced cholesterol metabolism disorders affect lamellar body membrane fluidity, impairing fusion with the plasma membrane and exocytosis of contents. Secreted tubular myelin requires structural reorganization to spread into the monolayer film needed to reduce surface tension, a process also susceptible to disruption by the inflammatory environment (e.g., leaked plasma proteins) in the alveolar space (116).

### 5.3 Abnormal mechanosensing and dysregulated repair

Alveoli undergo continuous stretching and retraction during the respiratory cycle. AEC2s sense these mechanical signals via the integrin-cytoskeleton system and mechanosensitive ion channels, regulating their proliferation and differentiation programs (117). Viral-induced cytoskeletal destruction and loss of junction proteins not only destroy barrier integrity but also impair the cell’s mechanosensing ability. Abnormal mechanical signals (e.g., uneven stretching due to local atelectasis) may transmit erroneous proliferation or transformation signals via mechanosensitive transcription factors like YAP/TAZ, promoting abnormal repair rather than functional regeneration. Under severe or persistent injury, pro-fibrotic pathways such as Wnt/β-catenin and TGF-β/Smad are aberrantly activated, leading to fibroblast activation and excessive extracellular matrix deposition, ultimately causing permanent alveolar structural remodeling, i.e., pulmonary fibrosis (118).

## 6 Evolution of model systems and future research paradigms

### 6.1 From static culture to dynamic physiological systems

Traditional 2D cell culture models fail to mimic the complex 3D structure and dynamic mechanical environment of the alveoli. Primary AEC2s cultured at the Air-Liquid Interface (ALI) can form polarized and functional alveolar epithelial models, allowing for more realistic apical viral infection simulations (119–121). Lung organoid technology, through stem cell self-organization, forms 3D structures containing multiple alveolar epithelial cell types, drastically improving cell-cell interactions and microenvironmental signaling, and has been successfully used to model IAV, SARS-CoV-2, and RSV infections (122–126). Advanced lung-on-a-chip systems integrate alveolar epithelium, endothelium, immune cells, and circulating fluids, and can apply physiological mechanical stretch, providing an unprecedented physiologically relevant platform for studying virus-host interactions and drug testing (127–129).

### 6.2 From population averages to single-cell resolution

AEC2 populations exhibit significant heterogeneity in response to viral infection. Single-cell RNA sequencing reveals distinct AEC2 subsets during infection, including those in strong antiviral states, metabolic remodeling states, stress-senescent states, and dying cells (130–132). Combining scATAC-seq to analyze chromatin accessibility allows mapping of the dynamic changes in transcriptional regulatory networks (133). Future research needs to integrate spatial transcriptomics and multiplex immunofluorescence imaging (134) to resolve the localization and signaling communication between immune cells and AEC2s in different states while preserving tissue spatial structure, deciphering the local microenvironmental signals driving disease progression or resolution (135).

### 6.3 From linear causality to network medicine

Based on the profound understanding of the aforementioned complex molecular networks, future host-directed therapies should shift towards network pharmacology strategies (136). This requires computational systems biology approaches to integrate multi-omics data and known molecular interaction networks, identifying key nodes or vulnerable edges within the network—targets that have the greatest impact on overall network stability and can be safely intervened against (137). For instance, simultaneously modulating multiple related pathways mildly (e.g., moderately inhibiting necroptosis while regulating metabolism) may be more effective and less toxic than potently inhibiting a single pathway. The application of AI and machine learning will accelerate the prediction and validation of optimal multi-target intervention combinations (138–140).

## 7 Conclusion and therapeutic perspectives

In summary, the comparative analysis of the pathogenic mechanisms of IAV, SARS-CoV-2, and RSV reveals a common paradigm transcending superficial differences: the severity of viral pneumonia is largely driven by the convergent destruction of core functional modules in specific alveolar epithelial cells (AEC2s). The strategies of these three viruses ultimately converge on three levels: (1) uncoupling organelle (mitochondria, ER) function from immune signaling; (2) disrupting programmed regulation of gene expression (post-transcriptional, epigenetic); and (3) manipulating the cell death balance towards viral dissemination and inflammation amplification.

**Figure 5:**
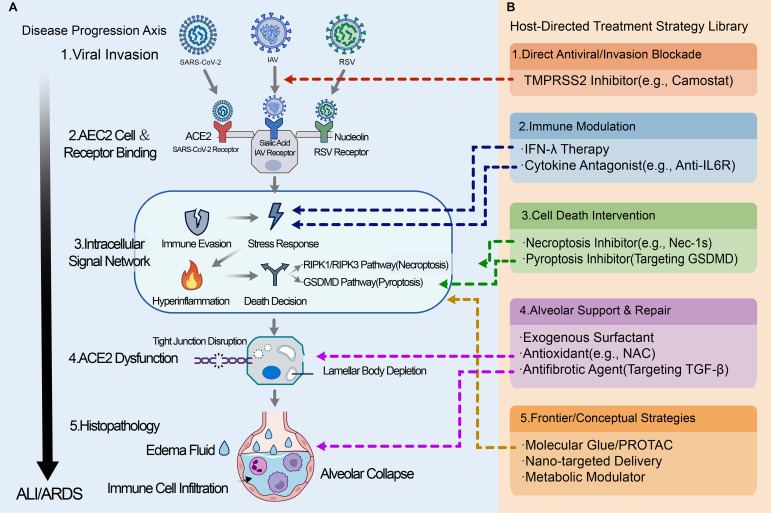
AEC2-centered disease-progression axis and host-directed treatment strategy library for convergent viral lung injury. **(A)** Disease progression axis (conceptual map): a staged AEC2-centered framework from viral invasion, AEC2 receptor engagement, and intracellular signaling disruption to AEC2 dysfunction (including tight-junction disruption and lamellar body depletion), histopathologic injury (edema fluid, immune-cell infiltration, alveolar collapse), and progression to ALI/ARDS. **(B)** Host-directed treatment strategy library: representative intervention classes are organized by the main process they are intended to target, including (1) direct antiviral/invasion blockade, (2) immune modulation, (3) cell-death intervention, (4) alveolar support/repair, and (5) frontier/conceptual strategies. Colored dashed connectors map each strategy class to the stage(s) or process node(s) in panel A where intervention is conceptually expected to have the greatest impact. These links indicate therapeutic targeting logic/intervention windows, not confirmed efficacy for all viruses or all disease stages. This figure is intended as an AEC2-centered integrative framework to organize host-directed options across IAV, SARS-CoV-2, and RSV. It is not a clinical treatment algorithm. Short-term/near-term strategies (e.g., antiviral entry blockade, immune modulation, and selected cell-death pathway inhibitors) are separated from frontier/conceptual approaches (e.g., PROTAC/molecular glue, nano-targeted delivery, metabolic modulators), which face translational constraints such as distal-lung delivery, onset kinetics in acute disease, and safety. Symbols: gray vertical arrow indicates disease progression toward ALI/ARDS; colored dashed connectors indicate conceptual intervention mapping between panel B strategy classes and panel A disease stages/processes. AEC2, type II alveolar epithelial cell; ALI, acute lung injury; ARDS, acute respiratory distress syndrome; IFN-λ, interferon lambda; IL-6R, interleukin-6 receptor; RIPK1/3, receptor-interacting protein kinase 1/3; GSDMD, gasdermin D; TMPRSS2, transmembrane serine protease 2; NAC, N-acetylcysteine; TGF-β, transforming growth factor beta; PROTAC, proteolysis-targeting chimera; IAV, influenza A virus; RSV, respiratory syncytial virus.

This synthesis suggests therapeutic entry points that align with shared hubs. **Figure 5** maps these options along the cascade from invasion to epithelial repair. We separate near-term approaches from longer-term platform strategies. Near-term host-directed options emphasize interventions with feasible delivery and rapid onset in acute disease. They include approaches that modulate interferon signaling, limit maladaptive inflammation, and reduce oxidative stress at the distal lung. Longer-term platform strategies aim for higher specificity. They include targeted protein degradation and targeted delivery systems. These approaches require careful evaluation of distal-lung delivery, onset kinetics in acute disease, and safety. Specifically, targeting the cell-death decision checkpoint (a convergent hub), Proteolysis Targeting Chimera (PROTAC) technology can be utilized to develop death pathway-specific PROTACs. Small molecules designed to simultaneously bind an E3 ubiquitin ligase (e.g., cIAP1) and a key necroptosis kinase (RIPK3) or pyroptosis executor (GSDMD) can induce their targeted degradation, theoretically precisely interrupting the virus-driven inflammatory death pathway while avoiding the side effects of broad apoptosis inhibition (141–143). Similarly, targeting viral immune evasion proteins (e.g., SARS-CoV-2 PLpro) with viral protein-specific PROTACs can remove them from infected cells, restoring the host ubiquitin signaling network (144, 145).

Furthermore, recognizing that the essence of viral pneumonia is driven by the dual engines of viral replication and host hyperinflammation, future PROTAC strategies can evolve towards dual-target or multi-target approaches (146–148). For example, a dual-target PROTAC molecule could bind an E3 ligase (e.g., CRBN) on one end, and on the other, bind both a key viral replication enzyme (e.g., SARS-CoV-2 PLpro or Mpro) and a host hyperactive inflammatory signaling hub (e.g., kinase IRAK4). This dual-targeting strategy aims to achieve dual efficacy: forced degradation of viral proteins to inhibit replication and precise suppression of uncontrolled innate immune signals to mitigate the cytokine storm. Compared to single-target inhibitors or their simple combination, dual-target PROTACs theoretically offer more synergistic, precise, and pharmacokinetically controllable therapeutic effects, representing a significant step from single-point intervention to network regulation in host-directed therapy. Additionally, given that mitochondrial oxidative stress (another convergent hub) suffered by AEC2s is a core link leading to their functional collapse, traditional systemic antioxidants (e.g., NAC) struggle to accumulate at the lesion site. Utilizing alveolar epithelium-targeting nanocarriers (e.g., surface-modified with peptides recognizing AEC2-specific receptors) (149–151), ROS scavengers (e.g., Mito-TEMPO) or metabolic modulators can be precisely delivered to damaged AEC2s and their mitochondria (152–154). This targeted nanocarrier strategy aims to correct the pathogenic metabolic-oxidative loop at the source, maximizing efficacy while minimizing systemic toxicity. It is worth noting that these frontier strategies face unique challenges in acute viral pneumonia application. For instance, matching the time window between PROTAC cellular permeability and the rapid viral life cycle; assessing enrichment efficiency and off-target risks of nano-targeted delivery systems in highly inflamed, edematous lung tissue; and the potential interference of metabolic modulators with energy homeostasis in other organs (155–157). Therefore, future research must not only validate the efficacy of these strategies in AEC2 models but also focus on solving their spatiotemporal precision—how to deliver therapeutic agents precisely to damaged AEC2s within the appropriate infection time window and achieve sufficiently rapid and reversible regulatory effects. This requires us to move beyond single molecular targets to the engineering design of the entire drug delivery and regulation system. With deepening understanding of molecular details and the rapid development of organoids, organs-on-chips, and AI technologies, developing broad-spectrum, highly effective therapies that mimic and enhance the natural resilience of the host defense system has become a feasible goal (158–160). Ultimately, a profound understanding of this sophisticated and complex molecular game between viruses and AEC2s will be key to winning the next battle against emerging respiratory viral pandemics.
